# Adaptation effects of medial forebrain bundle micro-electrical stimulation

**DOI:** 10.1080/21655979.2019.1599628

**Published:** 2019-04-12

**Authors:** Sepideh Farakhor, Vahid Shalchyan, Mohammad Reza Daliri

**Affiliations:** Neuroscience & Neuroengineering Research Lab., Biomedical Engineering Department, School of Electrical Engineering, Iran University of Science and Technology (IUST), Tehran, Iran

**Keywords:** Adaptation, navigation, reward area, rat, medial forebrain bundle

## Abstract

Brain micro-electrical stimulation and its applications are among the most important issues in the field of brain science and neurophysiology. Deep brain stimulation techniques have been used in different theraputic or alternative medicine applications including chronic pain control, tremor control, Parkinson’s disease control and depression control. Recently, brain electrical stimulation has been used for tele-control and navigation of small animals such as rodents and birds. Electrical stimulation of the medial forebrain bundle (MFB) area has been reported to induce a pleasure sensation in rat which can be used as a virtual reward for rat navigation. In all cases of electrical stimulation, the temporal adaptation may deteriorate the instantaneous effects of the stimulation. Here, we study the adaptation effects of the MFB electrical stimulation in rats. The animals are taught to press a key in an operant conditioning chamber to self-stimulate the MFB region and receive a virtual reward for each key press. Based on the number of key presses, and statistical analyses the effects of adaptation on MFB stimulation is evaluated. The stimulation frequency were changed from 100 to 400 Hz, the amplitude were changed from 50 to 170 µA and the pulse-width were changed from 180 to 2000 µs. In the frequency of 250 Hz the adaptation effect were observed. The amplitude did not show a significant effect on MFB adaptation. For all values of pulse-widths, the adaptation occurred over two consecutive days, meaning that the number of key presses on the second day was less than the first day.

## 1. Introduction

One of the important issues in the neurophysiology is the subject of deep brain stimulation and its widespread use. In this method of stimulation, electrical pulses are sent to specific points of the brain using surgical procedures by implanting the electrodes. The applications of this stimulation method include controlling chronic pains, tremor control, controlling Parkinson’s disease, controlling depression, and controlling animals remotely and directing them on a specific path. Given the great progress during the last decade in this field, the animals can be directed at a specific pathway by applying electrical stimulation to the specific region of the brain. Among the different known regions of the brain, many researchers have chosen to reward the dopaminergic pathway as a target area for stimulation. Dopaminergic pathways have strong links with dopaminergic neurons that connect the two regions of the brain. Dopaminergic neuron carries dopamine as a neurotransmitter in its synaptic destination. All researchers admit that stimulation of the MFB region results in rewards and pleasure, and the rewarding stimulation effect is regulated by the appropriate dosage of dopamine antagonists [1–3]. Since the activation of the mesolimbic pathway leads to a sense of satisfaction and rewards, many research groups use the excitation of this area to train the rat. However, in order to control and guide the animals, stimulation of this area is not enough on its own and should be accompanied with movement motivation.

**Table 1.** MFB micro-electrical stimulation parameters.

Pulses	10	20				
Frequency (Hz)	100	250	400			
Pulse width (µs)	180	350	500	1000	1500	2000
Pulse amplitude (µA)	90	130	170

**Figure 2.**
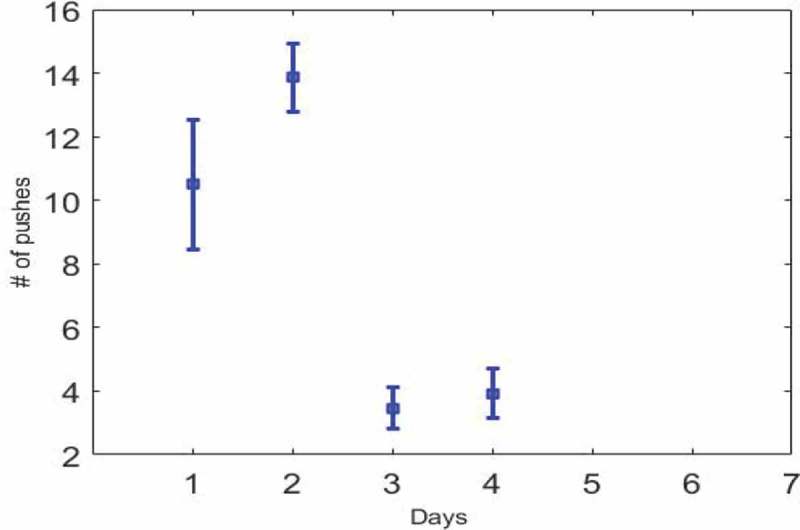
**Error bar chart**. The standard error is plotted for Figure 1, which shows a reduction on the third day and the rat’s unwillingness to press the key.

Conditioning chamber is designed for animal behavioral studies and can be used to teach the virtual reward sense in rodents by stimulating the MFB area [4]. Neural adaptation is a physiological phenomenon seen in many different sensory brain areas. This mechanism reduces the response of neuron to repetitive stimulation in a time period [5,6]. In most sensory systems, this function is still unknown. In the case of the adaptation of the MFB, it has been stated that rats attempted to end the self-stimulation they initially want it if the stimulation is prolonged [7,8]. Two theories attempted to explain this contradiction. The first type stated that the brain stimulation capacity changed from positive to negative. This change in drive capacity increases the negative component to the original positive component. According to this theory, the initial positive stimulus becomes unpleasant if prolonged. Of course, some of the theories in this group believe that rats can escape such a stimulus [8–11]. According to the second theory, if there was continuity of stimulation, less compliance is observed, and the rate of the response continues to decrease. As a result, rats discontinue self-stimulation to get rid of this adaptability and receive more rewards when triggering again. This theory expressed the adaptability process [12] and was consistent with the leakage integral model [13].

In studies on rat navigation, stimulation of a reward area like MFB is used to motivate the rat. Talwar et al. have shown for the first time that the animal can be remotely controlled by micro-stimulation of certain brain regions such as S1 and MFB [14]. In 2005, Hermer stated that one of the methods of rewarding is a conditioning chamber and applying electrical self-stimulation to the brain. In the training of the rat robot, the MFB stimulation was used as a reward and forward motion [15,16]. In another study, conducted by Sun in 2012, maze solution was performed only on the basis of MFB stimulation. Rats first were trained in a T-maze and the MFB stimulation was applied. When the rats chose the wrong path, the stimulation was discontinued and the rats discovered their mistakes [17]. There has been a lot of research on rat navigation in recent years [18,19] but there are not many studies on the MFB adaptation. In an article by Stein in 1962, explained two reasons for limiting reward stimulation. The first was the adaptation, which the effect of stimulation decreases over time and rats ended up triggering if dissatisfied. The second case is the unpleasantness, which is due to reward change to punishment, and rats tended to end the self-stimulation [12,20]. In one experiment, the animal was forced to control the duration of stimulation using the on/off lever in the Skinner box. In the results section, the reward theory suggested that the duration of long stimulation increased with increasing levels of current, as high intensities maintain a sense of satisfaction. Stein concluded that prolonged reward stimulation was sensitive to the electrode implanting area [11,21].

William Hods did a similar job, and implanted electrodes in various reward areas [22]. In 1991, the effect of the current on the maximum reward expressed, it was concluded that when the pulse frequency increases, the pulses sent to the MFB are saturated at a range of 200 to 361 pulses per second, and there is no increase more than that [23].

We used stimulation of the medial forebrain bundle (MFB) as ‘virtual’ rewards. The MFB stimulation was sent through the electrodes implanted in the brain. In a long time, the reward region is less susceptible to stimulation, that is to say, the response to the stimulus was reduced, and it may not be possible for the animal to be guided properly, and our attempt was to prevent the occurrence of reward adaptation. In this study, we investigated the important stimulation parameters such as the number of pulses, frequency, amplitude and pulse widths in a long time to find the parameters that adaptation occurred and to guide it by changing the parameters (increasing or decreasing) to avoid from MFB adaptation. We also obtained the parameters that were pressed too high in an operant conditioning chamber as the optimal stimulation parameters. First, with the surgery we implanted the electrode in the MFB area, after training the animal in an operant conditioning chamber, we got the number of times the key was pressed by the animal. Given the number of key presses and statitical analysis, we found the parameters of the adaptation.

The superiority of this study from previous studies is the issue of the MFB adaptation. In most cases, previous studies have investigated the duration of stimulation, but here, in addition to a comprehensive review of the individual parameters involved in electrical stimulation, the optimal parameters of stimulation were found.

## 2. Method

### 2.1. Subjects

Six adult male Wistar rats (200–300 g) were used. All the groups received MFB stimulation. All rats were housed individually with food and water ad libitum. All procedures used in the study were in accordance with the guide for the care and use of laboratory animals.

### 2.2. Stimulating electrodes

Stimulating electrodes were made from pairs of insulated Nichrome wires (A-M Systems, Formvar-Insulated Nichrome Wire, diameter: Bare 0.002 in., Coated 0.0026 in.). The Nichrome wires were twisted to form a bipolar electrode, with a 0.4 mm vertical separation between two tips, 0.3 mm of each tip was then exposed by peeling off the insulating formvar layer via sharply pointed cutter under a microscope. The impedance of the electrodes should be less than 100 KΩ. The electrodes impedance was around 30 KΩ.

### 2.3. Animal surgery

All rats were given at least 3 days to familiarize with the laboratory environment before surgical implantation. Briefly, the rat was anesthetized with xylazine hydrochloride (10 mg/kg) and ketamine hydrochloride (100 mg/kg). After tail pinching resulted in no movement, the rat was placed in a stereotaxic apparatus and bregma and lambda were adjusted to the horizontal level. The scalp was then locally numb with a subcutaneous injection of lidocaine. Next, the midline of the scalp was incised, the soft tissue on the skull surface was scraped. To further ensure the absence of additional tissues, Hydrogen Peroxide was used, which helps to clean the surface of the skull. five small burr holes were drilled into the skull for the placement of anchor screws and a ground electrode, stainless steel screws (tip diameter 1.0 mm) were then implanted into each hole. A hole was drilled into the skull (−3.8 mm posterior to the bregma, ±1.6 mm lateral to midline). The electrode was implanted vertically through the hole at the level of the lateral hypothalamus (8.2 mm below the dura) for stimulation of the medial forebrain bundle. So a craniotomy was made to permit introduction of the stimulating electrode to MFB (AP: −3.8, ML: ±1.6, DV: 8.2 mm) [24]. Of course, these values were for a standard animal. By measuring the distance between the lambda and bregma, we found the coordinates of the surgical rat. After electrode planting is done craniotomies were covered with dental acrylic. Following the completion of surgery, enrofloxacin (5 mg/kg) and meloxicam (0.2 mg/kg) were injected to the rat for 3 days after surgery to prevent pain and infection and each rat was housed separately in regular cages and given 7 days for full recovery [25,26].

### 2.4. Apparatus

An operant-conditioning chamber made of plexiglass (30cm×24cm×24 cm) was used in this study to optimize stimulation parameters. The chamber was equipped with two keys on its floor. With the push of one of the keys, stimulation was sent to the rat and the other key was not provoked. The key that received stimulation was attached to the Arduino board, which received the trigger applied through the trigger of the device and transferred to the key that was lit up LED and counted by pressing each key. The MFB stimulus was delivered via a stimulator (cerestim R96 micro-stimulator, BlackRock microsystems) that was triggered by a computer controlled by the stim manager. Training sessions began 7 days after surgery in an operant conditioning chamber. In MFB reward training, the rat is trained to press a key to obtain the MFB stimulus-reward, until it presses the key continuously to obtain the MFB stimuli after it has been placed before the key [27,28].

The animal was connected to the stimulator via wires. A key press delivered a stimulation train to the MFB (10 biphasic pulses with pulse width: 100 µs, pulse amplitude:50 µA, Frequency:100 Hz). The rats were screened for self-stimulation in the operant conditioning chamber. The rats were trained for 4 days for key pressure. Each rat was placed in an operant conditioning chamber about 30 minutes for 3 times every day. It was 2 hours between every 30 minutes, first rat number 1 was in the box after 30 minutes rat number 2 was placed in the chamber and continue until the end of the first session, then start the second and third session. We continue training until the number of pressures reaches a certain level, and after that, no stimulation is sent to the rats for two days. According to a recent study [29], MFB stimulation was applied every day 30 minutes to an hour for 2–10 days. Regarding the multiplicity of stimulation parameters and the wide range of each one and to prevent the fatigue and also the saturation of the stimulation parameters, MFB stimulation was planned as such. The rest time was considered to reduce the effect of previous stimulation parameter in each session.

One of the rats was removed from the experiments due to an increase in the impedance of the electrode. Two rats were introduced as a control group that tested after 2 days with the same parameters as before, to determine whether the decreasing trend was due to fatigue or adaptation to the stimulation.

The other three rats were tested with static parameters (10 biphasic pulses, pulse amplitude: 50 µA, pulse frequency: 100 Hz, and pulse width: 180, 260, 350 µs, with a distance of 60 minutes between the sessions. Each parameter was tested for 2 consecutive days. Every day rats tested for 3 sessions.

Again we test rats with (20 biphasic pulses, pulse amplitude:170 µA, pulse frequency:100 Hz, pulse duration: 500,1000,1500,2000 µs).

For another group of rats, 10 pulses with an amplitude of 50 μA and a pulse width of 100 µs, we changed the frequency parameter from 100, 250 and 400 Hz, and repeated the same experiment for two consecutive days in three 30-minutes sessions. After the change of each parameter, we gave rest to the rats for 2 days.

We have two groups for amplitude. The parameters of the first group are as follows: 20 biphasic pulses, frequency:100 Hz, pulse duration: 500 µs, pulse amplitude: 90,130,170 µA.

Second group are as follows: 20 biphasic pulses, frequency:100 HZ, pulse width: 1000 µs, pulse amplitude: 90,130,170 µA).  Changes in stimulus parameters are shown in Table 1.

We tested all the parameters for 2 consecutive days each day three times each time for 30 minutes.

By changing each parameter, the number of pressed keys was written. During the test, each group was also tested as a sham group so that the condition was the same, but no stimulation was applied.

To evaluate the test, we changed the location of the key and the placement of the rats in the groups to ensure that any chance of testing was achieved.

### 2.5. Statistic analysis

The Wilcoxon ranksum test is a nonparametric alternative to the two-sample *t*-test. P-values obtained in ranksum tests for statistically significant differences between key press of all rats in different stimulation conditions. The p-value less than 0.05 was considered as significant.

### 2.6. Histology

Following termination of the experiment, rats were perfused with normal saline followed by 10% formalin. The brains were then removed from the skull and sectioned to permit localization of the electrode tips.

## 3. Results

The purpose of the analysis is to investigate MFB adaptation over a long time, with the results obtained based on the number of pressed keys, we use statistical methods and charting the modified parameters that we have been changed.

### 3.1. The effect of stimulation on the control group

The purpose of the control group was that after four days of stimulation with fixed parameters and a two-day rest, the number of key pressure was reduced for fatigue or adaptability to reward stimulation. When prolonged stimulation is delivered at a high pulse frequency (≥100), the initial pulses contribute the most to the rewarding effect. Later pulses are affected by the reduced ability of the neurons or synapses to transmit signals along the neural network due to fatigue. When the neural network is becoming fatigued, each additional pulse contributes less to the rewarding effectiveness of the stimulation. When the durations of the stimulations are beyond the duration at which the neural network fatigues, the animal treats the stimulations equally. Therefore, the stronger stimulus will remain for a shorter amount of time [30].

We divided the rats into categories. We stimulated a group with the same previous parameters, which also reduced the number of pressed keys, but the other group, which increased the pulse width, increased the pressure of the keys compared to the previous one. This is not a result of fatigue and this theory was rejected, the reason for the reduction of pressures over time is an adaptation to reward stimulation. If this reduction was a result of fatigue, the key press should be increased after the rest period with the same parameters as before.

### 3.2. The effect of stimulation on the training group

We charted the number of key pressures for each session for each rat. With regard to the results of their lack of fatigue, this reduction can be seen in the adaptation of the reward area. By charting every four days for each rat, the number of pressures on the third day decreased and then rose again on the fourth day. This increase wasn’t impressive and the effects of adaptation may remain on the fourth day too. Although in the course of training it can’t be precisely nominated for adaptation, we can say that reduction is due to it.

**Figure 1.**
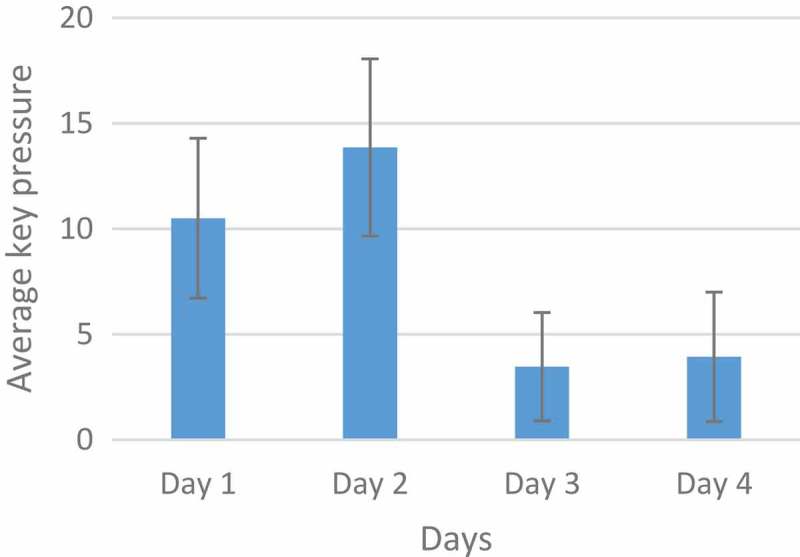
**An analysis of the training group**. The horizontal line represents the training days and the vertical line represents the average number of key presses in two days and three sessions every day. The decrease on the third day is due to the adaptation. The mean and the standard deviation bar of the key presses are shown in the figure.

Considering the concept of adaptation, the changes in key press should be checked over a long time. In Figure 1, the number of pressed keys per day is averaged for all rats, the reduction is clear on the third day. Also,  in Figure 2 the error bars plot on the third day, indicating the coherence of responses and the willingness of the rats to pressure the key. Regarding the ranksum test (p < 0.001) between the second and third day, there is a significant reduction that confirms the adaptation of reward stimulation. With respect to the third and fourth day (p = 0.7) it is clear that this adaptation has continued until the fourth day.

Our optimum point is the second day. Rats have given the most and the best response to electrical stimulation. So, in the navigation of the rats, the parameters expressed on the second day can be the best answer.

### 3.3. The effect of stimulation on the frequency group

**Figure 3.**
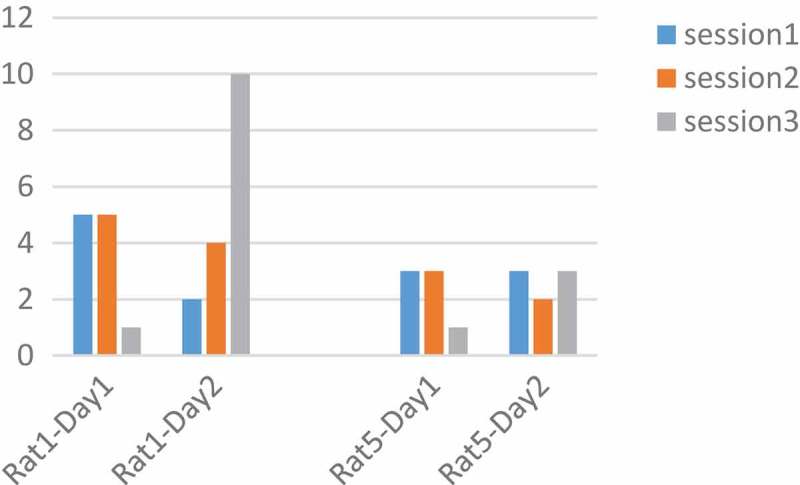
**Performance of rats at 100 Hz**. Two Rats No. 1 and 5 were tested for a frequency of 100 Hz, but they did not have a regular process during the days and sessions.

**Figure 4.**
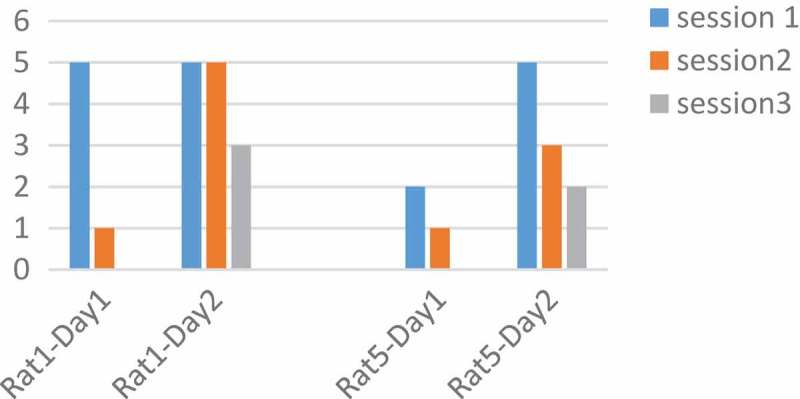
**Performance of rats at 250 Hz**. The number of pressed keys were reduced during the day and in three sessions. Adaptation occurred at a frequency of 250 Hz.

**Figure 5.**
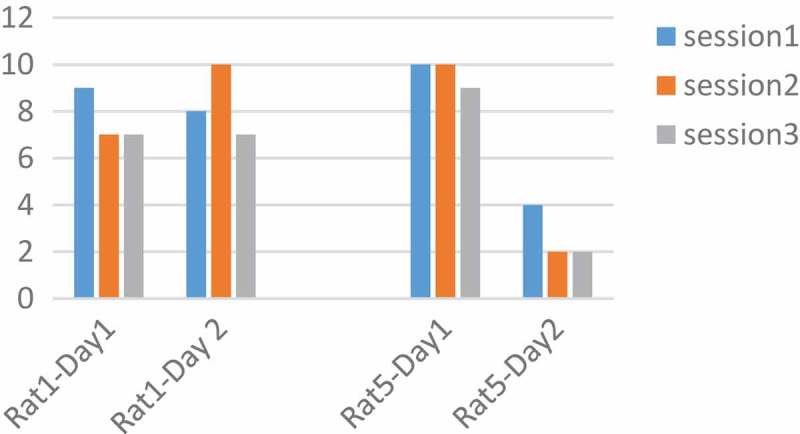
**Performance of rats at 400 Hz**. Two Rats No. 1 and 5 were tested for a frequency of 400 Hz, but according to the figure, they did not have a regular process during the days and sessions.

The frequency was tested at 100, 250, 400 Hz. According to the articles, with increasing frequency the tendency of the rat to press the key increases, and with increasing frequency, the number of pressures should also be increased [13,23]. Adaptation is significant over time. Therefore, the performance of both rats was plotted for frequency in two days and three sessions.  As shown in Figures 3,4 and 5 it is only at 250 Hz, that all rats have a downward trend during three sessions a day. The pressures were averaged over three sessions in two days. We saw a decrease in the frequency of 250 Hz and then the increase at a frequency of 400 Hz. This decrease implies the idea of MFB adaptation in a long time and the parameter that the adaptation occurred was 250 Hz.

**Figure 6.**
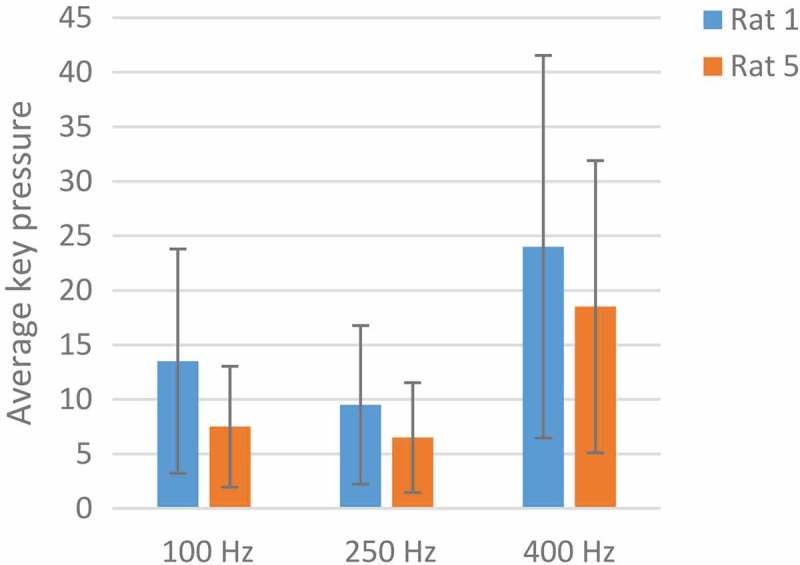
**An analysis of the optimum frequency**. The blue color indicates Rat No. 1 and the orange color indicates the Rat No 5 at three frequencies of 100, 250, and 400 Hz. The average key pressure in both rats dropped at 250 Hz, rising again at 400 Hz. The best response to stimulation was at 400 Hz.

With the conclusion of the p-value with the ranksum test, the p-values of less than 0.05 show significant changes. Increasing the key pressure from 250 Hz to 400 Hz in markedly determined by p-value <0.05(p = 0.03). In Figure 6 at 400 Hz, the rats pressed the keys more steadily and the number of pressed keys was higher. The optimum frequency for MFB stimulation was 400 Hz.

### 3.4. The effect of stimulation on the amplitude group

**Figure 7.**
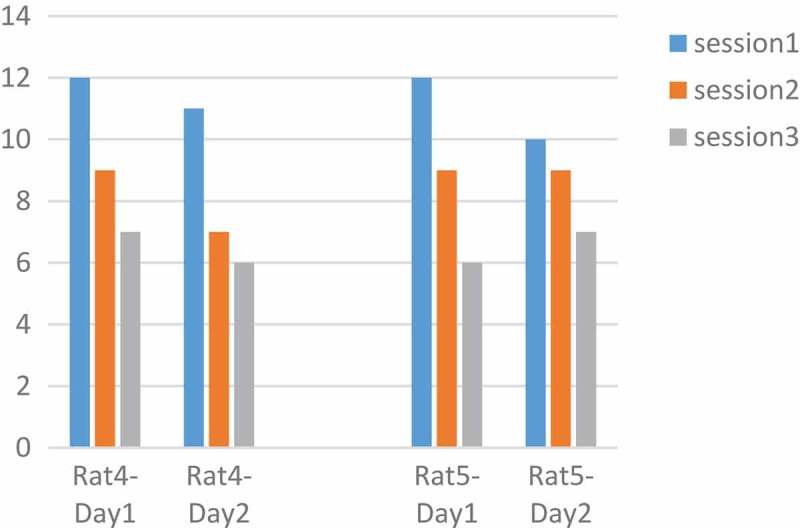
**Performance of rats at 90 µA**. Rats No 1,3 with 20 biphasic pulse, amplitude:90µ A, pulse width:500 µs, frequency: 100 Hz.

**Figure 8.**
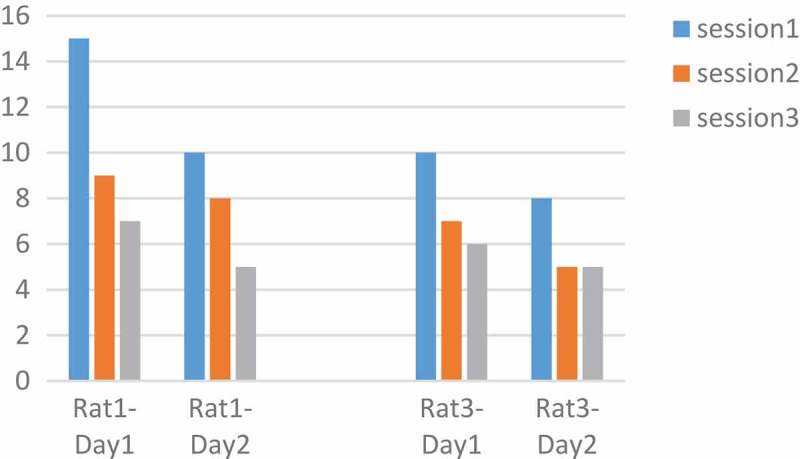
**Performance of rats at 130 µA**. Rats No 4,5 with 20 biphasic pulse, amplitude:130 µA, pulse width:1000 µs, frequency:100 Hz.

Rats number 1 and 3 were tested with a pulse duration of 500 µs and rats number 4 and 5 with 1000 µs. We plotted changes for each rat in two days and three sessions. The intensity and the adaptation will be the opposite, so as the intensity grows, the adaptation takes place over a longer period [31]. In Figure 7 rats No.1 and 3 at 90 μA and 500 µs had downward trend per day. Also, the number of keys pressed on the second day was less than the first day. But  in Figure 8, rats No.4 and 5 at 130 μA and 1000 µs had downward trend per day. Changes in other parameters were disorderly. However, by calculating the p-value and obtaining the p-value greater than 0.05 between two days of stimulation with the ranksum test, can be said that with these parameters and changing the amplitude, the adaptation does not occur and the amplitude changes do not have much effect. The occurrence of the adaptation was not related to a single parameter and all the parameters of the stimulation were involved.

**Figure 9.**
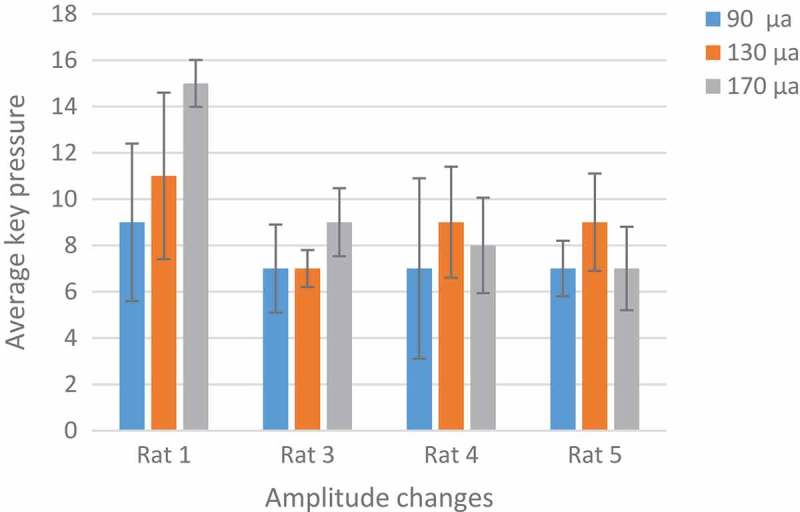
**Analysis of optimal amplitude**. Rat No.1,3 both are in increasing mood and there is no adaptation to stimulation but rat No.4,5 have a little decrease at 170 µA. the difference between the two groups is because of their different pulse width. 170 µA was an optimum stimulation parameter because rats press the key more than others.

In Figure 9, the number of keys pressed by the rat averaged in two days. 170 µA was an optimum stimulation parameter because rats press the key more than others. The animal’s desire for stimulation is high and this is very helpful in animal navigation.

### 3.5. The effect of stimulation on the pulse width group

**Figure 10.**
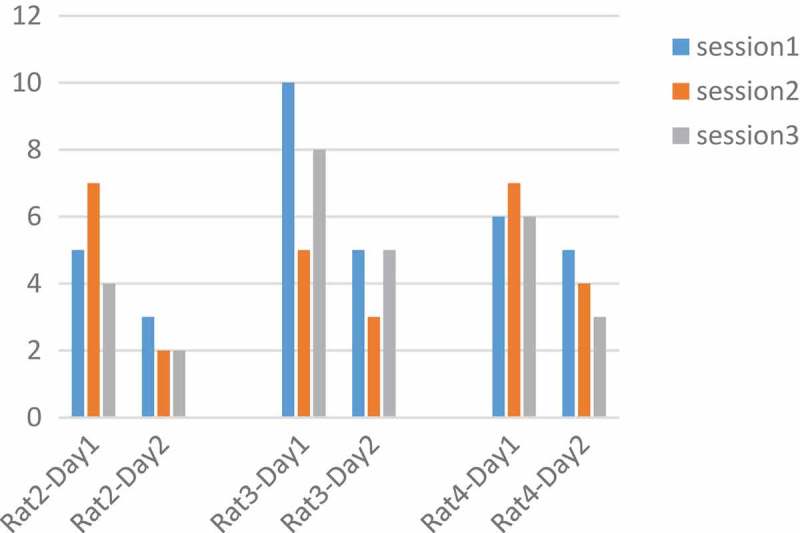
Performance of rats at 260 µs.

In the analysis of these rats, the number of key pressures was plotted in two days, the variation for rat 3 was 180, 260, 350, 500, 1500 µs and for rat 4 was 180, 260, 350, 1000, 2000 µs and for rat 2 180,260,350 µs and rat 5 1000, 2000 µs. Based on the articles, with increasing pulse durations, the desired increase to press the key [26]. The results obtained are not regular according to pulse width charts. Results are not the same for all rats. For example, within a pulse width of 180 µs and p = 0.003 at two days, rat number 4 in the second day was in a decreasing process. Similarly  in Figure 10, in 260 µs with p = 0.001, rats number 2 and 4 on the second day and 350 µs with p = 0.01 rat 2 on both days and 4 on the second day and 1000 µs rat number 4 on the second day and rat 5 on both days had signs of adaptation. But as mentioned, because the situation is not the same for all rats, it is not possible to make a conclusive result. The thing that is identical in all rats and in all the parameters is the low number of presses on the second day to the first. It may be concluded that adaptation occurred not in a day, but in between days.

**Figure 11.**
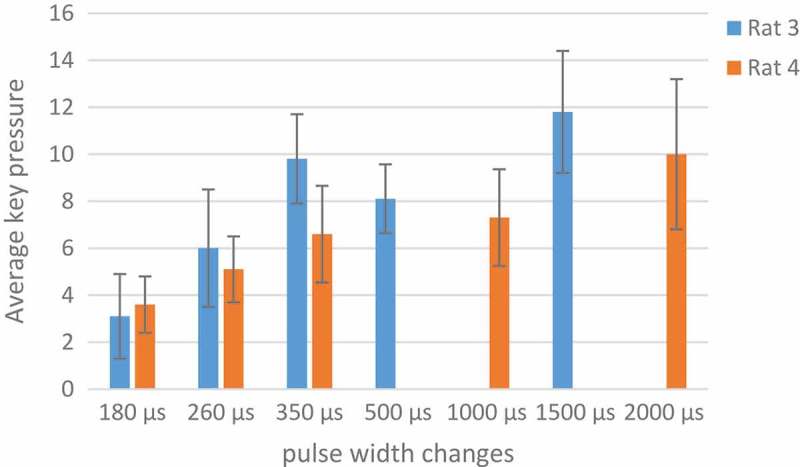
An analysis of optimum pulse width.

In Figure 11, key pressures averaged in two days. 1500 µs was the optimum pulse width because of rats high tendency to push the key.

### 3.6. The effect of stimulation on the sham group

During the experiment, all the groups tested as a sham group. The rats in this group were reluctant to press the keys because they did not receive a reward by pressing the key, so they refused to press the key or sit in the corner of the operant conditioning chamber. The number of key pressures in this group was mostly one in the first session and in other sessions don’t press the key. In fact, rats have no motivation for the push. This suggests that pressing the key was not a chance, and the rats were felt the reward stimulation.

## 4. Discussion and conclusion

In this study, we showed the adaptation of MFB reward area due to repeated electrical stimulation in a long time in five rats. This study is helpful to treat diseases such as Parkinson and navigate rats in order to find the injured people in natural disasters which cannot be passed through other animals and humans because of the high volume of debris. Rat robots have enough flexibility to cross the harsh and impassable places. Due to an adaptation makes the rat navigation difficult, we found adaptation parameters to prevent it from happening.

The superiority of this study from previous studies is that there is no interference with other results, and we completely and specifically mentioned to the issue of the MFB adaptation. In most cases, previous studies have been investigated the duration of stimulation, but in this project, in addition to a comprehensive review of the individual parameters involved in electrical stimulation, the optimal parameters of stimulation were found. The superiority of this study from previous studies is that there is no interference with other results, and we completely and specifically mentioned to the issue of the MFB adaptation. In most cases, previous studies have been investigated the duration of stimulation, but in this project, in addition to a comprehensive review of the individual parameters involved in electrical stimulation, the optimal parameters of stimulation were found.

In our experiment, all the parameters involved in the adaptation, such as the amplitude and frequency, and the pulse width and number of pulses were examined in the range that the stimulation device allowed. The lower the frequency, the lower the preference of the rat for the key pressure [13,23]. Adaptation and frequency also have a direct relation. In the case of pulse width, it can also be said that there is a direct relation to the adaptation, but the amplitude has an opposite relation with the adaptation, so the higher the amplitude, the more time it takes to adapt [31]. In general, by increasing each of the stimulation parameters, the rat’s incentive to press the key and receives a higher reward, and when it reaches the adaptation point, this number of pressed keys decreases and the increase in the stimulation parameter increases the number of key pressures again.

By designing the task and testing the results and drawing charts with the performance of every rat on each session and two days، adaptation to electrical stimulation was observed at the third day of training (P = 0.00009). By calculating the p-value (p = 0.7) of the third and fourth days we noticed that there is no significant difference, as a result, adaptation remain until the fourth day of stimulation. At 250 Hz with a p value = 0.03, the number of key pressures decreased and again increased at 400 Hz. With 10 biphasic pulses at 50 µA and 100 µs and 250 Hz, the MFB region adapted to stimulation. There’s no exact response for amplitude, maybe because of the opposite relation of amplitude and adaptation and higher amplitudes needed for adaptation. Due to the p > 0.05, no adaptation was observed in the results of the amplitude group. Because of p < 0.05 just 180,260,350,1000 µs were examined in results. Adaptation did not occur during a day for rats, but in all pulse widths, the number of pressures on day 2 was less than the first. To ensure the results, the sham group was also tested and the keys place was changed in the chamber, and the placement of the rats was also random.
